# PATIENT AND CARE PARTNER VALUES WHEN CONSIDERING DEMENTIA TREATMENT OUTCOMES

**DOI:** 10.1093/geroni/igae098.4042

**Published:** 2024-12-31

**Authors:** Maayra Butt, Josiah Drakes, Paola Rosa, Melanie Bahti, Kristin Harkins, Jason Karlawish, Scott Halpern, Catherine Auriemma

**Affiliations:** University of Nevada Las Vegas, Mechanicsburg, Pennsylvania, United States; University of Pennsylvania, Philadelphia, Pennsylvania, United States; University of Pennsylvania, Philadelphia, Pennsylvania, United States; University of Pennsylvania, Philadelphia, Pennsylvania, United States; University of Pennsylvania, Philadelphia, Pennsylvania, United States; University of Pennsylvania, Philadelphia, Pennsylvania, United States; University of Pennsylvania, Philadelphia, Pennsylvania, United States; University of Pennsylvania, Philadelphia, Pennsylvania, United States

## Abstract

We sought to understand how persons living with dementia (PWD) and their family care partners value hypothetical treatment outcomes relative to one other using a point allocation task. Participants distributed 100 points across six potential outcomes based on perceived relative importance. Outcomes included slowed decline in brain function, reduced agitation and combativeness, reduced family caregiver stress, prolonged ability to perform activities of daily living, increased time living at home, and increased socialization and engagement. Equality of distributions between outcome scores and between subgroups were compared using Wilcoxon signed-rank and rank-sum tests, respectively. 201 individuals participated (n=154 (77%) care partners and n=47 (23%) PWD). Across all participants, slowing declines in brain function scored highest (20, IQR 10, 30, p< 0.05 for all pair-wise comparisons). Care partners compared to patients more highly valued reduced agitation and combativeness (15.5, (IQR 10, 25) vs 10 (IQR 3, 16), p< 0.001). Patients compared to care partners more highly valued reducing family caregiver stress (16 (IQR 10, 30) vs 10 (IQR 5,20), p=0.003). Among care partners, those whose loved one had mild dementia more highly valued slowing declines in brain function (20 (IQR 10, 37.5) vs 15 (IQR 10, 25), p=0.02). Conversely, care partners of those with moderate or severe dementia more highly valued reducing agitation and combativeness (20 (IQR 10, 25) vs 12.5 (7.5, 21) p=0.007). PWD and dementia care partners expressed differing values when considering treatment outcomes. The development of future treatments should consider the most valued outcomes and disease severity to enhance potential treatment satisfaction.

